# From fractionation to financials: economic and clinical implications of hypofractionation in German outpatient radiotherapy practice

**DOI:** 10.1007/s00066-025-02484-y

**Published:** 2025-11-26

**Authors:** Anastassia Löser, Monika Huth, Akvile Juskeviciute, Tina Peters, Anne-Sophie Mehdorn, Charlotte Flüh, Moritz Bültmann, Oksana Zemskova, Larysa Liubich, Alexander von Ohlen, Cedric Carl, Lorenz Hahn, Alla Smagarynska, Dirk Rades, Christian Schmidt

**Affiliations:** 1 Department of Radiotherapy, University Medical Center Schleswig-Holstein Campus Lübeck, Ratzeburger Allee 160, 23562 Lübeck, Germany; 2 Department of Anesthesiology and Intensive Care Medicine, University Medical Center Hamburg-Eppendorf, Hamburg-Eppendorf, Germany; 3 Department for Corporate Development, Process Management, Kiel Municipal Hospital, Kiel, Germany; 4 Department of General Surgery, Visceral, Thoracic, Transplantation and Pediatric Surgery, University Medical Center Schleswig-Holstein Campus Kiel, Kiel, Germany; 5 Department of Neurosurgery, University Medical Center Göttingen, Göttingen, Germany; 6 Department of Anesthesiology and Intensive Care Medicine, Martin Luther Hospital, Berlin, Germany; 7 State Institution Romodanov Neurosurgery Institute, National Academy of Medical Sciences of Ukraine, Kyiv, Ukraine; 8 PricewaterhouseCoopers GmbH Auditing company, Hamburg, Germany; 9 Center for Radiotherapy, Strahlentherapie Nord, Bremen Gröpelingen, Germany; 10 Practice for Radiology, Radiotherapy and Nuclear Medicine, Kassel, Germany; 11 LifeLink Medical GmbH, Düsseldorf, Germany

**Keywords:** Cost-effectiveness, Prostate cancer, Breast cancer, Value-based healthcare, Reimbursement models

## Abstract

**Background and objective:**

Thie study aimed to examine the economic implications of different radiotherapy fractionation schemes, specifically normofractionation (NF) and hypofractionation (HF), for breast and prostate cancer in the outpatient setting of the German healthcare system. In times of workforce shortages, limited machine availability, and rising patient numbers, the study aims to identify which fractionation approach offers the highest value in terms of efficiency and economic sustainability, aligning with a value-based healthcare framework.

**Methods:**

Economic models were developed using German reimbursement data (EBM), treatment costs, machine usage, and realistic patient volumes. Three breast cancer fractionation schemes (conventional NF with 30 fractions, i.e., 25 fractions to the whole breast +5 boost fractions), NF with simultaneous integrated boost (SIB) comprising 28 fractions, and HF with 20 fractions (15 fractions to the whole breast +5 boost fractions) as well as two prostate cancer regimens (39 × 2.0 Gy versus 20 × 3.0 Gy) were compared. A standardized clinic setup with two linear accelerators and defined full-time staff was assumed. Analyses included cost, break-even points, contribution margins, and personnel needs in both scenarios (HF and NF).

**Results:**

Despite lower reimbursement per case, HF regimens yielded significantly higher economic efficiency due to increased patient throughput and reduced staff-time per treatment. Over 10 years, the total revenue per linear accelerator for HF breast cancer treatments reached approximately € 56.9 million, compared to € 40.2 million and € 46.6 million for the two NF approaches. A one-time investment of € 50,000 for implementing HF (e.g., for software, training, and workflow optimization) could be amortized within a few days, depending on the scenario. Simulation models further demonstrated substantial efficiency gains under hypofractionation without the need to expand machine capacity—an important strategy amidst staffing shortages and increasing demand.

**Conclusion:**

When supported by efficient clinic organization and sufficient patient volume, HF offers clear economic advantages over traditional fractionation schemes. However, for widespread implementation, structural reform of the current outpatient reimbursement system is desirable.

**Supplementary Information:**

The online version of this article (10.1007/s00066-025-02484-y) contains supplementary material, which is available to authorized users.

## Introduction

Radiation oncology (RO) remains an important cornerstone of modern cancer care, utilized in roughly half of all oncological treatment cases [1, 2]. Technological advancements—such as image-guided planning, high-precision linear accelerators (LINACs), and the integration of artificial intelligence (AI)—have significantly enhanced the efficacy and safety of radiotherapy. However, these innovations have also increased operational complexity and financial demands. The acquisition and maintenance of advanced equipment, coupled with a growing shortage of qualified staff, particularly radiation therapy technologists (RTTs), pose substantial challenges to both outpatient and inpatient RO services [3].

In Germany, about 85% of radiation treatments are delivered in an outpatient setting [1]. The increasing demand for radiotherapy, driven by an aging population and rising cancer incidence, exacerbates existing infrastructure and staffing bottlenecks [1, 4, 5]. According to forecasts by Thun et al. and the World Health Organization (WHO), global cancer cases are expected to rise to 35 million annually by 2050 [5]. Concurrently, there is a critical shortage of RTTs in Germany, with 46% of hospitals reporting difficulties in staffing [3].

For several tumor entities, including breast and prostate cancer, it has been shown that hypofractionation (HF) is noninferior to normofractionation (NF). The HypoG-01 phase III UNICANCER trial included 1221 patients with breast cancer (T1–3 N0 M0) who were randomized into two treatment arms (15 × 2.67 Gy versus 25 × 2.0 Gy). An average of 12 regional lymph nodes were removed in 82.8% of patients. During a median follow-up period of 4.8 years, there were no significant differences in the occurrence of lymphoedema (primary endpoint: time to occurrence of lymphoedema). Therefore, the authors concluded that moderate HF was noninferior to NF, even though locoregional node irradiation was included [6]. The FAST-Forward trial, a phase III, randomized, noninferiority study, aimed to further reduce the duration of radiation therapy in early breast cancer. It compared a five-fraction radiation therapy regimen over 1 week to moderate HF (15 fractions over 3 weeks), demonstrating noninferiority in terms of local tumor control and normal tissue toxicity (up to 5 years after the end of radiation) [7].

A widely used moderate HF concept in prostate cancer is based on the results of the CHHiP trial, a randomized phase III study. Here, prostate cancer patients (pT1b-T3a N0 M0) were randomized 1:1:1 into an NF (37 × 2 Gy up to a cumulative dose of 74 Gy) or an HF arm (either 20 × 3 Gy or 19 × 3.0 Gy). A total of 3216 men were included in the CHHiP trial. Already in the year of publication (2016), the authors concluded that HF with 20 × 3.0 Gy was noninferior to the NF variant in terms of recurrence-free survival and can therefore be recommended as the new gold standard [8]. In the PACE‑B trial, a phase III study in patients with localized prostate cancer (T1–T2 prostate cancer, Gleason ≤ 3 + 4, PSA ≤ 20 ng/mL), stereotactic radiotherapy (SBRT) consisting of five fractions was compared with a conventionally fractionated and a moderately hypofractionated radiation variant. The authors concluded that ultrahypofractionation (UHF) was noninferior to the control arm (conventional or moderate HF) in terms of biochemical control [9].

In addition to the clinically evident data situation for certain tumor entities, several authors from non-German healthcare systems have listed HF as an economically attractive alternative to NF [10, 11]. Moore et al. demonstrated that moderate HF for prostate cancer can reduce treatment costs by 25–50% [12]. The economic logic, as previously described by Lievens, is straightforward: the cost of radiation therapy is directly linked to overall treatment time, which is a function of both the number of fractions and the time required per session [13].

In Germany, clinical decision-making is influenced by a fragmented reimbursement framework: outpatient care is governed by the *einheitlicher Bewertungsmaßstab* (German uniform value scale, EBM) system [14], which reimburses each fraction individually, while inpatient care is funded through case-based diagnosis-related groups (DRGs) [15]. Especially in the outpatient setting, NF appears to be more profitable because each additional session generates extra revenue (per patient). A switch to HF could lead to financial shortfalls unless centers can increase their patient volumes. Additionally, the transition to HF involves upfront investments in advanced technology, staff training, and expanded quality assurance protocols.

This analysis aimed to evaluate the economic implications of different radiotherapy fractionation schemes (particularly HF versus NF) by examining their cost-effectiveness in the German outpatient setting (EBM-based reimbursement) for breast and prostate cancer patients. Furthermore, it explores the potential of HF to improve healthcare efficiency within the framework of value-based care.

## Materials and methods

This study was conducted as a model-based economic analysis of a prototypical German radio-oncology (RO) department operating within the framework of the national outpatient reimbursement system (EBM) [14]. The model represents a mid-sized facility staffed by seven board-certified radiation oncologists, six RTTs, five medical physicists, and four administrative personnel. This department treats patients on two LINACs for 8.5 h a day in single-shift operation.

**Fig. 1:**
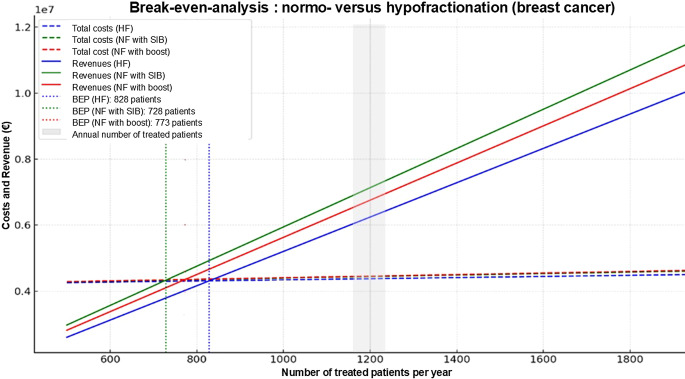
Break-even analysis for breast cancer. Graphical representation of the break-even points (*BEP*) for the three radiation concepts investigated for breast cancer (hypofractionation with 20 fractions and normofractionation with 30 or 28 fractions). It should be noted that in reality, the annual case numbers of approximately 1200 patients are composed of different tumor entities (i.e., not exclusively breast cancer treatments). *HF* hypofractionation, *NF* normofractionation, *BEP* break-even point, *SIB* simultaneous integrated boost, *Fx* fractions, *Pat.* patients

For our model case, we assumed an annual number of approximately 1200 patients. This figure reflects the total number of patients across all tumor entities, based on median structural data, and was chosen to provide a standardized framework for economic modeling. We acknowledge that this number does not correspond to tumor-specific caseloads for breast or prostate cancer.

Our model does not directly mirror either a typical outpatient practice or a university hospital department. Rather, it represents a compromise between smaller private practices and larger academic centers. We acknowledge that staffing patterns can vary substantially between treatment centers, and the results of our analysis may therefore differ under alternative prerequisites.

### Main objective

The objective was to assess the economic implications and capacity effects of adopting HF schedules compared to conventional NF, without assuming additional investment costs specific to HF technology. For doing this, the present analysis focused on two common cancer indications with established evidence for both NF and HF radiotherapy:

- non-metastatic breast cancer following breast-conserving surgery for adjuvant whole-breast irradiation with or without endocrine therapy and
- localized or locally advanced prostate cancer receiving definitive external beam radiotherapy in curative intent, with or without androgen deprivation therapy (ADT), but excluding elective nodal irradiation.

### Cost assessment

The cost model differentiated between fixed and variable cost components. Fixed costs included salaries of medical and nonmedical staff as well as depreciation of major equipment (e.g., linear accelerators, planning CT) including annual maintenance cost and facility overhead. Moreover, we included additional fixed nonpersonnel costs associated with quality assurance infrastructure and software licenses. All fixed costs were allocated proportionally based on departmental structure and assumed full annual operation at standard capacity.

Variable costs included medical consumables, maintenance and energy consumption per fraction, information technology (IT), and administrative workload per treatment session.

Personnel costs accounted for the largest portion of fixed expenses and were calculated based on current public sector wage agreements in Germany. Salaries were determined using the relevant pay scales for each professional group [16]: radiation oncologists were remunerated according to TV‑Ä, pay group Ä2, level 3; medical physicists were assigned to TV‑L, pay group E14, level 3; RTTs were classified under TV‑L, pay group E9a, level 3; and medical and administrative staff salaries were based on TV‑L, pay group E5, level 3. These salary scales were sourced from official tariff tables and reflect gross annual compensation, including standard contractual benefits but excluding overtime or shift bonuses [16].

Publicly accessible data and benchmarks were used to estimate costs, including reports from the German Hospital Institute (DKI), professional society publications (e.g., DEGRO (Deutsche Gesellschaft für Radioonkologie e.V., German Society for RO)), statutory reimbursement catalogs, and manufacturer specifications. All monetary values were standardized to reflect approximate 2024/2025 price levels.

### Calculation of treatment revenues

Treatment revenue was calculated using the EBM [14], where each radiotherapy session is reimbursed separately in the outpatient setting. The model compared conventional NF (breast cancer: 25 × 2.0 Gy plus 5 × 2.0 Gy boost or 28 fractions for breast cancer with simultaneously integrated boost, SIB; prostate cancer: 39 × 2.0 Gy fractions) with moderate HF (15 fractions à 2.67 Gy for breast cancer plus 5 × 2.0 Gy boost and 20 × 3.0 Gy for prostate cancer), based on current national and international clinical guideline recommendations [17, 18]. While the focus of this work was on comparing moderately and conventionally fractionated concepts, we have included the revenues for UHF concepts in the Supplementary Material. A BEP for UHF concepts was also calculated (e.g., PACE‑B or FAST-Forward) [7, 9].

This structural model did not include capital investments explicitly associated with implementing HF (e.g., image-guided radiotherapy or motion management systems), in order to isolate the effects of the choice of fractionation regimen under current infrastructural conditions. Since there are no standardized metrics in (U)HF to capture aspects of higher requirements for intellectual and cognitive resources in a reproducible manner, these factors were not explicitly included in our model. We acknowledge that these factors are particularly relevant for HF and UHF regimes, where precision and treatment complexity increase.

### Break-even analysis

To carry out a break-even analysis, the fixed and variable costs of the radio-oncology department presented were determined. This break-even analysis was conducted to determine the minimum number of patients required per year to cover all departmental costs (break-even point, BEP):

$$BEP=\frac{\textit{Fixed}\,\textit{costs}}{\textit{Revenue}\,per\,\textit{patient}-\textit{variable}\,\textit{costs}}$$

### Further considerations

#### Patient number dynamics

To estimate how patient numbers need to increase over time to compensate for HF regimens (under a reference NF scenario with 1200 patients/year) we applied three different formulas: 

1) The total revenue (TotalRevenue_ref_) under the reference scenario was calculated by means of the following formula:

$$\begin{aligned}&\textit{TotalRevenue}_{\textit{ref}}\\ &=1{,}200 \times R_{\textit{ref}} (\textit{revenue}\,per\,\textit{patient}\textit{in\,the}\,\textit{reference}\,\textit{regimen}) \end{aligned}$$

2) The required number of patients (N_required_) to be treated with an alternative treatment regimen (R_alt_) to result in the same revenue was defined by

$$\textit{N}_{\textit{required}}=\frac{\textit{TotalRevenue}\left(ref\right)}{R\left(alt\right)}.$$

3) Then the following formula was used to calculate the absolute and relative (%) increase in patient numbers (*N*):

$$\Updelta N=\textit{N}_{\textit{required}}-1{,}200,and{\%}\,\textit{ increase }=\frac{\Updelta N}{1{,}200}x100{\%}.$$

#### Impact of hypofractionation on RTT staffing requirements

To estimate the staffing requirements for RTTs, the time expenditure per patient was calculated based on the number of fractions administered. A treatment time of 10 min per fraction (equivalent to 0.1667 h) was assumed, covering patient setup, positioning, and beam-on time. Based on a typical annual patient volume of 1200 cases, the total yearly working hours required for various fractionation schemes (e.g., HF with 20 fractions vs. NF with 28 or 30 fractions) were determined. The resulting annual total workload was compared to the standard annual working time of a full-time RTT, which is 1694 h (assuming a 38.5-hour workweek over 220 working days). The required full-time equivalents (FTEs) were then calculated by dividing the total workload by the available working time per full-time employee.

### Statistical analysis

**Fig. 2:**
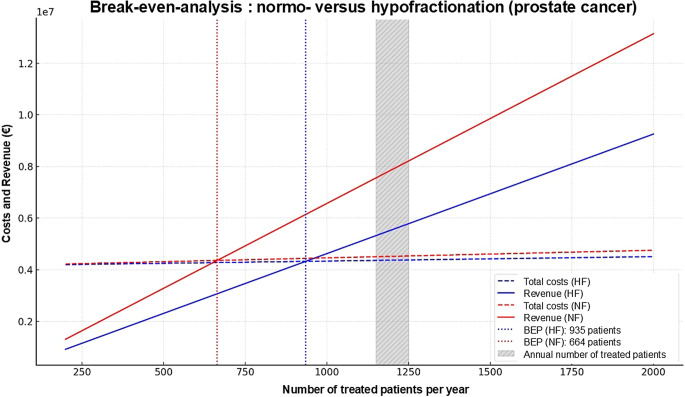
Break-even analysis for prostate cancer. Graphical representation of the break-even points (BEP) for the two radiation concepts investigated for prostate cancer (hypofractionation with 20 fractions versus conventional normofractionation with 39 fractions). *HF* hypofractionation, *NF* normofractionation, *BEP* break-even point, *Fx* fractions, *Pat.* patient

All calculations and economic modeling were performed using Microsoft Excel (Microsoft Corp., Redmond, WA, USA). The tool was used to simulate treatment volumes, calculate fixed and variable costs, and compare economic outcomes between conventional NF and HF regimens. Standard Excel functions and self-developed spreadsheet formulas were applied to model annual personnel costs, treatment throughput, and potential revenue under the German outpatient reimbursement system (EBM). Figs. 1 and 2 demonstrating the results of break-even analysis were created using ChatGPT (version 4.0, 2025). For language editing, the large language model ChatGPT (OpenAI, San Francisco, CA, USA) was applied.

No inferential statistical methods were applied, as the analysis was designed as a model-based economic evaluation rather than an empirical or clinical study.

## Results

### Calculation of revenue and cost assessment

The German outpatient reimbursement system (EBM) was consulted to determine the revenues in the outpatient sector for the fractionation concepts examined for breast and prostate cancer. The detailed breakdown is attached in the Supplementary Material. Briefly, HF irradiation of the breast (20 fractions including boost) in Germany can generate € 5073.69, conventional NF irradiation (30 fractions in total) € 5371.14, and NF irradiation with SIB € 5,815.24. For prostate cancer, HF (20 × 3.0 Gy) generates € 4628.90, while conventional NF (39 × 2.0 Gy) generates € 6441.96. In comparison, a UHF treatment regimen consisting of five fractions leads to revenues of € 1822.83 for both tumor entities.

In a radiotherapy department, fixed costs represent a significant component of the overall cost structure. Personnel costs make up the largest share. In contrast to variable costs, which increase directly with the number of patients treated or radiation fractions applied, fixed costs remain constant over a certain period (e.g., 1 year) regardless of the actual capacity use. Table 1 lists the fixed costs (model operation of two LINACs).

**Table 1:** List of fixed costs of a model radiotherapy department

	Basis for assessment	Annual costs
*Amortization of equipment and technology*		
Linear accelerator	Linear depreciation/acquisition on January 1 at a purchase price of € 2.5 million (in each case per linear accelerator); € 312,500 × 2 = € 625,000	€ 625,000
Useful life: 8 years/straight-line depreciation rate: 12		
CT for radiation planning incl. storage equipment and contrast agent injector	Linear depreciation/acquisition on January 1 at a purchase price of € 400,000	€ 50,000
Useful life: 8 years/linear depreciation rate: 12	–	
Software (including planning systems, dose calculation)	Multiple workstations, modules, ≥ 5 licenses, planning programs for IMRT/VMAT, etc.	€ 1,000,000
Maintenance costs (usually via maintenance contract)	Full maintenance contract (for two linear accelerators)	€ 600,000
*Personnel costs (incl. social security contributions)*		
RO	TV-Ä/Ä2/level 3	€ 1,813,920.99
Medical physicists/experts (MPE)	TV-L/E14/level 3	
RTT	TV-L/E9a/level 3	
Other (medical assistants, administration staff)	TV-L/E5/level 3	
*Buildings and infrastructure *[17]		
Rent or depreciation of the practice/clinic rooms	Building already depreciated or no rent payable	€ 0
Structural radiation protection	Conversion costs incl. radiation protection bunker (structural radiation protection) = € 4,000,000	€ 80,000
Depreciation over 50 years (2% per year) in accordance with Section 7 (4) German Income Tax Act		
*Total sum*		*€* *4,168,920.99*

This model represents a mid-sized facility staffed by 7 board-certified radiation oncologists, 6 RTTs, 5 medical physicists, and 4 administrative personnel. This department treats patients on two LINACs for 8.5 h a day in single-shift operation. The calculation of the collective agreements refers to the latest version valid in 2025. Useful lives and depreciation rates were determined based on tables published by the German Federal Ministry of Finance.

*RTT* radiotherapists, *CT* computer tomography, *RO* radiation oncologists, *TV* collective wage agreement (German: *Tarifvertrag*)

In addition to the annual fixed costs, the estimated variable costs per single irradiation fraction were also determined. The individual factors are summarized in Table 2. The variable costs amount to € 171.92 for the irradiation of breast and prostate cancer as part of HF irradiation (20 irradiation fractions). For breast cancer, NF radiation with 28 radiation fractions and SIB incurs variable costs of € 223.92 per patient, with € 236.92 per patient with 30 radiation fractions. A conventional NF prostate irradiation consisting of 39 irradiation sessions leads to variable costs of € 295.42. In the case of UHF (5 fractions), the variable costs per patient are significantly lower (€ 74.42).

**Table 2:** Estimated variable costs

		Basis for assessment	Costs
Electricity consumption of the appliances (30 cents/kwh)	LINAC	18 kW/h [18], 8.5 h patient operation, 15.5 h not completely switched off	€ 6.5^a^
	CT for radiation treatment planning	Variable (depending on scanning time and standby mode); average 10 kw/h	
	Other medical equipment (e.g., dose measurement, imaging, cooling and ventilation systems with 8‑fold air exchange per day, temperature control ft he rooms)	Estimated 20 kWh per day	
Consumables per patient	Thermoplastic masks for head and neck patients: € 50–150 per mask	€ 100 per thermoplastic radiation mask (e.g., for head and neck tumor irradiation)	€ 100^b^
	Fixation material/positioning (€ 20,000; used by 10,000 patients; renewal after 5 years [pads] or after approx. 7 years [positioning aids])	Multiple use: € 20,000/10,000 patients = € 2	€ 2^b^
	Dose-measuring strips/ionization chambers for quality assurance and other phys. equipment (incl. measuring phantoms, small-field chambers, etc.; according to manufacturer, generally useful life of 10 years)	Multiple use (10 years ≈ 12,000 patients); total price: € 467,000	€ 38.92^b^
Maintenance and wear	Included in the manufacturer’s maintenance contract	–	€ 0
Others	Overtime/bonuses for personnel	Conversion to compensatory time off (no payout)	€ 1^b^
	Cleaning and hygiene material (gloves, etc.)		
*Total sum*	*Variable costs per patient (without thermoplastic radiation mask)* *=* *€* *41.92* *+* *€* *6.5* *×* *number of applied fractions*		

*LINAC* linear accelerator

^a^Per fraction

^b^Per patient

### Break-even analysis

The calculation of the BEP is shown below for breast cancer with three different fractionation concepts:

- HF radiotherapy (20 fractions to the whole breast plus five sequential boost fractions):

$$BEP=\frac{\mathrm{\texteuro}4{,}168{,}920.99}{\mathrm{\texteuro}5{,}629.18-\mathrm{\texteuro}236.92}=850.5\,\textit{patients}$$

- Conventional NF radiotherapy (25 fractions to whole breast plus five sequential boost fractions):

$$BEP=\frac{\mathrm{\texteuro}4{,}168{,}920.99}{\mathrm{\texteuro}5{,}371.14-\mathrm{\texteuro}236.92}=812\,\textit{patients}$$

- NF radiotherapy (28 fractions to the whole breast including simultaneous-integrated boost):

$$BEP=\frac{\mathrm{\texteuro}4{,}168{,}920.99}{\mathrm{\texteuro}5{,}815.24-\mathrm{\texteuro}223.92}=745.6\,\textit{patients}$$

The same calculation method was used for prostate cancer: this resulted in BEPs of 935.4 and 678.3 for 20 and 39 fractions, respectively. Figures 1 and 2 illustrate the results of the break-even analysis. The annual patient number of 1200 patients is shaded gray.

The possibility of increasing the number of patients depends directly on the number of fractions applied and the total treatment time. If fewer fractions are applied (HF), more patients can be treated at the LINAC with the same resources. In the case of UHF (five fractions), the variable costs per patient are significantly lower (€ 74.42), with a corresponding BEP of 2384 patients.

The number of fractions per patient is lower with HF radiation. Fewer fractions lead to a shorter overall treatment time. Assuming that each patient is treated for an average of 10 min at the LINAC (including the necessary preparation and follow-up times), 20 fractions result in a total treatment time of 20 fractions × 10 min = 200 min (3.33 h); NF irradiation with 28 fractions and 30 fractions would result in a total treatment time of 280 min (4.67 h) and 300 min (5 h), respectively. Conventional NF irradiation of prostate cancer with 39 fractions results in a total treatment time of 390 min (6.5 h).

As part of the single-shift operation, patients are treated on the LINAC for a total of 8.5 h per day. Extrapolated for 220 working days per year, this means that 1870 working hours per year are spent at a department’s LINAC.

Table 3 summarizes the revenue situation (per linear accelerator) for breast and prostate cancer for the selected fractionation concept.

**Table 3:** Annual revenue situation for each fractionation scheme

	Breast cancer			Prostate cancer	
	HF	NF (inc. SIB)	NF	HF	NF
Number of Fx	20	28	30	20	39
Overall annual treatment time per LINAC (h)	1870	1870	1870	1870	1870
Total treatment time per fraction at the LINAC (mins)	10	10	10	10	10
Total treatment time at the LINAC per patient (min)	200	280	300	200	390
Maximum number of patients per year	561	401	374	561	288
Revenue/patient case (€)	5073.69	5371.14	5815.24	4628.90	6441.96
Maximum annual revenue (= max. number of patents × revenue/case; €)	*2,846,340.09*	*2,152,292.53*	*2,174,899.76*	*2,596,812.90*	*1,853,302.34*

*HF* hypofractionation, *NF* normofractionation, *SIB* simultaneous-integrated boost, *LINAC* linear accelerator, *Fx* fraction

On the one hand, the lowest number of patients is required for NF (as shown by different BEPs). This statement is valid for both tumor profitability levels. On the other hand, if there is a sufficient influx of patients who require radiotherapy, HF would be more advantageous by increasing the number of patients, as it allows more patients per year to receive radiation. This has a direct impact on the annual revenue situation of a radiation oncology department. Therefore, HF has a positive impact on the maximum annual revenue, which is superior to NF (Table 3).

Although NF is better remunerated per patient according to the EBM catalog, the total turnover increases with HF due to an increase in capacity, so that an outpatient radiation oncology department benefits from HF in the long term. Table 4 shows the economic superiority of HF over a timeframe of 10 years.

**Table 4:** Calculated revenue for 10 years

		Fx	Total treatment time per pat. (h)	Total working hours LINAC (10 years))	Lifetime pat. (10 years)	Revenue per pat. (€)	Total turnover (€)
*Breast cancer*	HF	20	3.3	37,400	11,218	5073.69	56,915,418.72
	NF	30	5.0	37,400	7479	5371.14	40,168,093.58
	NF	28	4.7	37,400	8013	5815.24	46,595,675.72
*Prostate cancer*	HF	20	3.3	37,400	11,218	4628.90	51,925,872.83
	NF	39	6.5	37,400	5753	6441.96	37,058,635.04

Cumulative turnover after 10 years for hypo- and normofractionation

*Pat.* patient(s), *HF* hypofractionation, *NF* normofractionation, *Fx* fraction, *LINAC* linear accelerator

### Further considerations

#### Patient number dynamics

To quantify how much patient throughput would need to grow to offset lower per-patient revenues under HF, we modelled our reference institution treating 1200 patients/year with common NF regimens (30 and 28 fractions) and calculated the number of patients required under alternative regimens to preserve the same total annual revenue.

For breast cancer (given an NF reference scenario of 30 fractions and a revenue of € 5371.14) a switch to HF (20 fractions; € 5073.69) would yield the following:

$$\textit{TotalRevenue}_{\textit{ref}}=1{,}200\times \mathrm{\texteuro}5371.14=\mathrm{\texteuro}6445,37$$

Therefore, the required number of patients with HF (20 fractions) would be approximately 1270 patients ($\frac{\mathrm{\mathrm{\texteuro}}6445{,}37}{\mathrm{\mathrm{\texteuro}}5073.69})$, meaning that an absolute increase of + 70 patients (≈ + 6.0%) would be necessary. A switch from the same NF to an UHF regimen (5 × 5 Gy; € 1822.83) means that 2536 patients and therefore an absolute increase of 2336 patients (+ 194.7%) would be required to maintain an institution’s total revenue. If the reference is a 28-fraction SIB regimen (€ 5815.24), the same HF (20 fractions) change requires + 14.6% (≈ 1375 patients).

For prostate cancer, replacing a 39-fraction NF regimen (€ 6441.96) with a 20-fraction HF regimen (€ 4628.90) would require ≈ + 39% (+ 470 patients) more patients (≈ 1670). By contrast, switching to UHF five-fraction schedules (example revenue € 1822.83/patient) would require very large increases in throughput (+ 3041 patients or + 253%).

#### Required equipment

The equipment required for the introduction of HF concepts entails minimal investments per center (e.g., enhanced quality assurance measures). The investments are primarily related to organizational and digital infrastructure, which are necessary to ensure patient safety even in shortened radiation therapy regimens. Table 4 shows a difference in annual revenue of + € 16.3 million for breast cancer treatments if HF with 20 fractions is used over 10 years (or 1200 patients) instead of 30 fractions. Even if a one-time investment of € 50,000 (e.g., for training) were necessary to introduce HF, this investment would pay off in less than 1 week:

Payback period for an investment of € 50,000:

$$\frac{\mathrm{\texteuro}50{,}000}{\mathrm{\texteuro}16{,}264{,}887}\approx 0.00307\,\textit{years}\approx 1.12\,\mathrm{days}$$

#### Impact on personnel requirements

The introduction of HF can effectively increase the number of patients per year (see above). For both medical and medical physics staff, this means an increase in workload (increased number of patient care and planning sessions).

Each radiation session on the linear accelerator is accompanied by an RTT. Our results show that HF can be used to conserve RTT staff resources (potential savings in times of staff shortages; Table 5).

**Table 5:** Annual RTT working hours for hypo- and normofractionation

	Number of Fx	Working hours/per patient	Working time/year (for 1200 patients; h)
HF	20	3.334	4000.8
NF	28	4.6676	5601.1
NF	30	5.001	6001.2
NF	39	6.5013	7801.6

Summary of RTT workload for each fractionation regimen. With 1200 treated patients per year, cumulative working time is lowest for hypofractionation.

*HF* hypofractionation, *NF* normofractionation, *Fx* fraction, *RTT *radiation therapy technologist

At German public and university hospitals, a full-time RTT position is a collectively agreed 38.5-hour week. With 220 working days/year (excluding staff absence due to sick days) and a 5-day week, there are 44 working weeks. Multiplying the 44 working weeks by a weekly working time of 38.5 h results in 1694 h per year for a full-time RTT position. The following personnel requirements (required MTR full-time positions) can be derived from this: assuming that an HF concept requires 4000.8 RTT working hours to treat 1200 patients, 2.4 RTT full-time positions would be required in our model. In comparison, NF concepts would require 3.3 full-time positions (28 fractions for each of these patients) and 3.5 full-time positions if each patient is treated 30 times.

## Discussion

The current analysis explores the economic impact of introducing HF in outpatient radiation oncology under conditions of staff shortages and limited resources. The analysis compares NF versus HF using two common cancer types—breast and prostate cancer—within a model radiation therapy unit.

The choice of the “right” fractionation scheme plays a fundamental role in radiotherapy treatment planning. While NF was considered the gold standard for decades, HF and even UHF have increasingly established themselves as equivalent or even superior alternatives in recent years [6–9, 19, 20]. It should be emphasized that our analysis primarily focused on treatment slot utilization and reimbursement effects. Therefore, it is important to acknowledge that HF and, in particular, UFH regimens (e.g., SBRT) are associated with increased intellectual and personnel requirements. The integration of advanced techniques such as molecular imaging for target definition, simultaneous integrated boost (SIB) approaches, and focal dose escalation requires substantial expertise from radiation oncologists, medical physicists, and RTTs. Radiobiological modeling, precise contouring, and rigorous treatment planning are indispensable for the safe implementation of higher doses per fraction. Moreover, additional monitoring of patients treated with (U)HF is necessary. These factors lead to increased personnel time and costs, which were not explicitly accounted for in our present cost models. Consequently, the assumption that all capacities freed through HF can be linearly reallocated to additional patients may overestimate the actual throughput gains. Therefore, future economic analyses should integrate these additional intellectual and personnel requirements, particularly when considering UHF treatment concepts.

Data on the economy and cost-effectiveness of HF compared to NF are very limited. The French working group led by Le Bras et al. evaluated the use of accelerated partial breast irradiation (20 fractions, twice a day, over 1 week of treatment) with standard and HF [21]. These authors examined insurance-based data over a 3-year horizon, while the measured parameters included so-called QALYs (quality-adjusted life years), i.e., the years of life spent in health (benefit of a medical intervention on life expectancy and quality of life). A QALY difference between the two randomized arms could not be shown. However, it must be emphasized that there was no explicit comparison between HF and NF [21]. In contrast, the French PROFIT study aimed to compare the cost-effectiveness between moderate HF and NF concepts in prostate cancer [22]. The authors of the PROFIT study also compared differences in QALYs and costs between the two radiation arms for prostate cancer (20 × 3.0 Gy versus 39 × 2.0 Gy). The estimated costs (€) from 2020 were combined with the QALYs to obtain an incremental cost-effectiveness ratio (ICER). This ratio compares the economic value of an intervention between two different therapies. Although the costs of HF were lower (€ 3,062) than for conventional NF (€ 4,285), the QALYs of HF were slightly higher. However, this observation lacked statistical significance. Recorded costs in the PROFIT study included transportation costs (> 50% of all costs examined), costs for doctors’ appointments, and costs for medical procedures including diagnostics and hospital stays [22]. Nevertheless, the results of the PROFIT trial are not directly comparable to our results: while the PROFIT study focused on an extrinsic approach, where the total costs of the respective healthcare system were set in relation to health and quality of life, the aim of the current study was to pursue an intrinsic economic approach to the economic advantages and disadvantages that arise for the individual radiotherapy treatment unit. However, the fact that HF (regardless of the tumor entity to be treated) at least results in lower revenues per patient but is also associated with lower overall health economic costs appears logical and comprehensible.

In Germany, the reimbursement of outpatient radiotherapy has so far largely been organized on a fraction-based basis—for example in the EBM via GOPs (*Gebührenordnungspositionen*: fee schedule items) such as 25321, 25324, or 25343 [14]. This leads to an inherent economic conflict: while HF concepts with fewer fractions could make medical care more efficient, the per capita revenue—at least in the short term—generally falls in proportion to the reduced number of fractions. However, our results show that from a business perspective, HF radiation concepts have a higher benefit-to-cost ratio than conventional NF radiation concepts. Assuming a corresponding patient influx, HF results in a higher annual turnover and leads to better utilization of equipment capacities. If the radiation equipment (including LINAC) is already available, the one-off investment costs appear to be comparatively low. Also, HF offers clear advantages in terms of personnel and resource efficiency, particularly in view of existing capacity bottlenecks, for example due to a shortage of RTTs or limited LINAC slots in Germany. To conclude, the present analysis demonstrates that the current EBM system in Germany does not promote the introduction of such HF concepts: fraction-based billing penalizes a lower number of treatment sessions and thus leads to an economic disadvantage for outpatient facilities. Innovative, efficient care is thus devalued economically, and a conflict of objectives arises between efficiency of care and financial stability.

It should be acknowledged that private billing schemes (GOÄ) and selectively reimbursed private patients (PP) may impact the economic role of HF in German radiotherapy. In contrast to the uniform reimbursement structure of the statutory health insurance system (EBM), private billing often allows higher per-fraction remuneration and may therefore mitigate the relative financial disadvantages of HF regimens. However, institutions usually treat a mixed patient population, which may partially offset the reduced reimbursement associated with fewer fractions and improve the overall financial sustainability of HF. Although precise statistics on the distribution of patients receiving radiation therapy who are covered by statutory or private health insurance are lacking, it can be assumed that most radiotherapy treatments in Germany are provided under statutory insurance. Therefore, the impact of GOÄ-based reimbursement remains limited. Nevertheless, private billing highlights the broader issue that reimbursement models directly shape clinical decision-making and that more flexible, cycle-based remuneration systems could better accommodate innovative fractionation schemes across both statutory and private care settings.

Despite the well-founded economic consideration of different fractionation concepts (HF vs. NF), the present analysis has some limitations that restrict its informative value and international comparability. First, this analysis is based on economic indicators that are only used in Germany due to its outpatient remuneration system (EBM). No regional differences (e.g., in patient structure or technical equipment) could be taken into account. Some assumptions, e.g., on the level of investment, equipment utilization, or number of fractions, are based on modeling. These are useful for an initial orientation, but do not reflect the full complexity of everyday clinical practice. Any fluctuations in variable costs (electricity prices or personnel costs), equipment downtimes, or different billing paths were not considered. In particular, it can be assumed that personnel costs are higher than assumed in this model.

The present analysis focused exclusively on the economic perspective in radiation oncology. Accordingly, no patient-centered aspects such as treatment preferences, travel distances, or psychological stress (e.g., due to more frequent fractions) were considered. These factors could provide valuable insights into the context of structured surveys or qualitative studies and supplement economic decisions. Although we assume that the choice of fractionation concept influences our patients’ quality of life, this was not recorded as part of the present analysis (no QALYs were determined). Therefore, it is not possible to draw a complete value-based healthcare comparison in the sense of international health economic standards. In particular, benchmarking with studies published elsewhere (e.g., from England or France) [21, 22], in which QALYs were included in the decision-making process, is not methodologically admissible.

In conclusion, HF in the German outpatient reimbursement system can generate more revenue by increasing patient numbers, although adequate outpatient billing structures for UHF still need to be created. In the future, adapting reimbursement structures to correctly reflect the clinical and economic value of HF will be essential to ensure sustainable, efficient, and patient-centered radiotherapy.

## Supplementary Information

Table 6 – Overview of fixed personnel costs by occupational group (according to collective wage agreement for German university hospitals).
Table 7 – Comparison of the revenue situation with four different fractionation concepts for breast cancer.
Table 8 – Revenues for hypofractionated (20 fractions and 5 fractions, respectively) and normofractionated (39 fractions) regimen for prostate cancer.
