# Supplementary material for: From fractionation to financials: economic and clinical implications of hypofractionation in German outpatient radiotherapy practice
Source: Strahlenther Onkol. 2025 Nov 26;202(3):319–29. doi: 10.1007/s00066-025-02484-y (PMC12953385; doi:10.1007/s00066-025-02484-y)
Supplement: Supplementary file 1 — Table 6 – Overview of fixed personnel costs by occupational group (according to collective wage agreement for German university hospitals). Table 7 – Comparison of the revenue situation with four different fractionation concepts for breast cancer. Table 8 – Revenues for hypofractionated (20 fractions and 5 fractions, respectively) and normofractionated (39 fractions) regimen for prostate cancer. [file 66_2025_2484_MOESM1_ESM.docx]

**Table 6 – Gross wage costs for various occupational groups in a single-shift operation with two linear accelerators**

| **5-day week** | **RO (TV-Ä)/TDL (level 3) (40 hours per week)** | **RTT (TV-L, E9a, level 3) (38.5 hours per week)** | **Medical physicist (TV-L, E14, level 3) (38.5 hours per week)** | **Medical assistant, accounting clerk, etc. (TV-L, E5, level 3) (38.5 hours per week)** | **Sum** |
| --- | --- | --- | --- | --- | --- |
|  | € 8596.06 | € 3818.66 | € 5662.24 | € 3330.99 |  |
| + pension insurance (9.3%) | € 799.43 | € 355.14 | € 526.59 | € 309.78 | € 1990.94 |
| + unemployment insurance (1.3%) | € 111.75 | € 49.64 | € 73.61 | € 43.30 | € 278.30 |
| + health insurance (7.3%) | € 627.51 | € 278.76 | € 413.34 | € 243.16 | € 1,562.78 |
| + nursing care insurance (1.8%) | € 154.73 | € 68.74 | € 101.92 | € 59.96 | € 385.34 |
| + accident insurance (1.5%) | € 128.94 | € 57.28 | € 84.93 | € 49.96 | € 321.12 |
| Gross monthly salary incl. social security contributions | € 10,418.42 | € 4628.22 | € 6862.63 | € 4037.16 | € 25.946,44 |
| Annual gross amount incl. social security contributions | € 125,021.10 | € 55,538.59 | € 82,351.62 | € 48,445.92 | € 311,357.22 |
| Hourly wage (gross) | € 60.11 | € 27.73 | € 41.11 | € 24.19 | € 153.13 |
| Daily wage (gross), 8 hours (+0.5 hours lunch break) | € 480.78 | € 213.58 | € 316.69 | € 186.30 | € 1197.34 |
| Number of employees/shift | 7 | 6 | 5 | 4 | 22 |
| Gross annual salary (incl. social security contributions) for all employees | € 875,147.68 | € 333,231.55 | € 411,758.09 | € 193,783.67 | **€ 1,813,920.99** |

Table 6 – Übersicht über die fixen Personalkosten je nach Berufsgruppe

RO = Radiation oncologist; RTT = Radiotherapist; TV = Collective wage agreement (Tarifvertrag);

| **Table 7 – Revenue for different fractionation concepts for breast cancer *(descriptions left in German for better comprehensibility)*** | | | | **HF: 15 x 2,667 Gy + 5 x 2,0 Gy Boost** | | | **NF: 25 x 2 Gy + 5 x 2,0 Gy Boost** | | **NF (SIB): 28 x 1,8 Gy incl. 28 x 0,3 Gy** | | **UHF: 5 x 5 Gy** |
| --- | --- | --- | --- | --- | --- | --- | --- | --- | --- | --- | --- |
| **Main plan** | | | | | | | | | | | |
| **EBM-**  **number** | **Description** | **Credits** | **Amount (€)** | **Factor** | | **Product (€)** | **Factor** | **Product (€)** | **Factor** | **Product (€)** | **Product (€)** |
| 25211 | Konsiliarpauschale bei bösartiger Erkrankung (Aufklärungsgespräch & Arztgespräch nach Neueinstellung) | 1041 | 129.02 | 2 | | 258.04 | 2 | 258.04 | 2 | 258.04 | 129.02 |
| 34360 | CT-gesteuerte Untersuchung von Organabschnitten für die Bestrahlungsplanung bei Tele- oder Brachytherapie | 354 | 43.87 | 1 | | 43.87 | 1 | 43.87 | 1 | 43.87 | 43.87 |
| 25342 | Rechnerunterstützte Bestrahlungsplanung für die perkutane Bestrahlung mit individueller Dosisplanung für irreguläre Felder mit individuellen Blöcken, Viellamellenkollimator, nicht koplanaren Feldern und/oder 3-D-Planung | 4744 | 587.94 | 1 | | 587.94 | 1 | 587.94 | 1 | 587.94 | 587.94 |
| 25343 | Zuschlag zur Gebührenordnungsposition 25342 für die rechnerunterstützte Hochpräzisionsbestrahlungsplanung (IMRT und/oder fraktionierte Stereotaxie), je Bestrahlungsserie | 1245 | 154.30 | 1 | | 154.30 | 1 | 154.30 | 1 | 154.30 | 154.30 |
| 25328 | Zuschlag bei Überschreitung der Einzeldosis ≥ 2,5 Gy bei bösartiger Erkrankung, Zuschlag zur Gebührenordnungsposition 25321 bei Überschreitung der Einzeldosis ≥ 2,5 Gy, je Bestrahlungssitzung | 480 | 59.49 | 15 | | 892.35 | 0 | 0 | 0 | 0 | 297.45 |
| *To be continued on the next two pages* | | | | | | | | | | |  |
| **EBM-**  **number** | **Description** | **Credits** | | **Amount (€)** | **Factor** | **Product (€)** | **Factor** | **Product (€)** | **Factor** | **Product (€)** | **Product (€)** |
| 25321 | Bestrahlung mit einem Linearbeschleuniger bei bösartigen Erkrankungen oder bei raumfordernden Prozessen des zentralen Nervensystems, für das **erste** Zielvolumen, je Bestrahlungssitzung | 960 | | 118.98 | 15 | 1784.7 | 25 | 2,974.50 | 28 | 3331.44 | 594.9 |
| **Boost** | | | | | | | | | | | |
| 25342 | Rechnerunterstützte Bestrahlungsplanung für die perkutane Bestrahlung mit individueller Dosisplanung für irreguläre Felder mit individuellen Blöcken, Viellamellenkollimator, nicht koplanaren Feldern und/oder 3-D-Planung | 4744 | | 587.94 | 1 | 587.94 | 1 | 587.94 | 1 | 587.94 | 0 |
| 25343 | Zuschlag zur Gebührenordnungsposition 25342 für die rechnerunterstützte Hochpräzisionsbestrahlungsplanung (IMRT und/oder fraktionierte Stereotaxie), je Bestrahlungsserie | 1245 | | 154.30 | 1 | 154.30 | 1 | 154.30 | 0 | 0 | 0 |
| 25324 | Zuschlag bei mehr als einem Zielvolumen bei bösartiger Erkrankung | 241 | | 29.87 | 0 | 0 | 0 | 0 | 28 | 836.36 | 0 |
| 25321 | Bestrahlung mit einem Linearbeschleuniger bei bösartigen Erkrankungen oder bei raumfordernden Prozessen des zentralen Nervensystems, für das erste Zielvolumen, je Bestrahlungssitzung | 960 | | 118.98 | 5 | 594.9 | 5 | 594.9 | 0 | 0 | 0 |
| **Breast cancer** | | | | | **15 x 2,667 Gy + 5 x 2,0 Gy Boost** | | **25 x 2 Gy + 5 x 2,0 Gy Boost** | | **28 x 1,8 Gy incl. 28 x 0,3 Gy (SIB)** | | **UHF: 5 x 5 Gy** |
| **EBM-**  **number** | **Description** | **Credits** | | **Amount (€)** | **Factor** | **Product (€)** | **Factor** | **Product (€)** | **Factor** | **Product (€)** | **Product (€)** |
| **Other** | | | | | | | | | | |  |
| 40110 | Kostenpauschale für die Versendung bzw. den Transport eines Briefes und/oder von schriftlichen Unterlagen |  | | 0.96 | 2 | 1.92 | 2 | 1.92 | 2 | 1.92 | 1.92 |
| 40111 | Kostenpauschale für die Übermittlung eines Telefaxes |  | | 0.05 | 1 | 0.05 | 1 | 0.05 | 1 | 0.05 | 0.05 |
| 01601 | Individueller Arztbrief | 108 | | 1338 | 1 | 13.38 | 1 | 13.38 | 1 | 13.38 | 13.38 |
|  |  |  | |  | **Sum** | **5,073.69** | **Sum** | **5,371.14** | **Sum** | **5,815.24** | **1,822,83** |

Table 7 – Comparison of the revenue situation with four different fractionation concepts for breast cancer. The comparison includes conventional fractionation regimen consisting of 25 fractions (main plan) + 5 boost fractions, conventional fractionation with simultaneous integrated boost (SIB) (a total of 28 fractions and correspondingly two target volumes, which are irradiated in parallel) and a hypofractionated variant, which consists of 20 irradiation sessions including the boost applied. Since ultra-hypofractionation is not a standard concept at most centers, the corresponding column on the right is shown in gray font.

EBM = uniform evaluation scale (Einheitlicher Bewertungsmaßstab); SIB = simultaneous integrated boost; IMRT = intensity-modulated radiotherapy; CT = computer tomography; HF = Hypofractionation; NF = Normofractionation; UHF = Ultrahypofractionation

**Table 8 – Revenue for different fractionation concepts for intermediate-risk prostate cancer *(descriptions left in German for better comprehensibility)***

| **Moderate HF: 20 x 3 Gy** | | | | | | **NF: 39 x 2 Gy (56 Gy main plan + 22 Gy boost)** | | | | **UHF:**  **5 x 5 Gy** |
| --- | --- | --- | --- | --- | --- | --- | --- | --- | --- | --- |
| **Main plan** | | | | | | | | | | |
| **EBM-**  **number** | **Description** | **Credits** | **Amount (€)** | **Factor** | **Product (€)** | **Credits** | **Amount (€)** | **Factor** | **Product (€)** | **Product (€)** |
| 25211 | Konsiliarpauschale bei bösartiger Erkrankung (Aufklärungsgespräch) | 1041 | 129.02 | 1 | 129.02 | 1041 | 129.02 | 1 | 129.02 | 129.02 |
| 34360 | CT-gesteuerte Untersuchung von Organabschnitten für die Bestrahlungsplanung bei Tele- oder Brachytherapie | 354 | 43.87 | 1 | 43.87 | 354 | 43.87 | 1 | 43.87 | 43.87 |
| 25342 | Rechnerunterstützte Bestrahlungsplanung für die perkutane Bestrahlung mit individueller Dosisplanung für irreguläre Felder mit individuellen Blöcken, Viellamellenkollimator, nicht koplanaren Feldern und/oder 3-D-Planung | 4744 | 587.94 | 1 | 587.94 | 4744 | 587.94 | 1 | 587.94 | 587.94 |
| 25343 | Zuschlag zur Gebührenordnungsposition 25342 für die rechnerunterstützte Hochpräzisionsbestrahlungsplanung (IMRT und/oder fraktionierte Stereotaxie), je Besrahlungsserie | 1245 | 154.30 | 1 | 154.30 | 1245 | 154.30 | 1 | 154.30 | 154.30 |
| 25328 | Zuschlag bei Überschreitung der Einzeldosis ≥ 2,5 Gy bei bösartiger Erkrankung, Zuschlag zur Gebührenordnungsposition 25321 bei Überschreitung der Einzeldosis ≥ 2,5 Gy, je Bestrahlungssitzung | 480 | 59.49 | 20 | 1,189.80 | 480 | 59.49 | 0 | 0 | 297.45 |
| *To be continued on the next two pages* | | | | | | | | | |  |
| **Moderate HF: 20 x 3 Gy: 20 x 3 Gy** | | | | | | **NF: 39 x 2 Gy (56 Gy main plan + 22 Gy boost)** | | | | **UHF:**  **5 x 5 Gy** |
| **EBM-**  **number** | **Description** | **Credits** | **Amount €)** | **Factor** | **Product (€)** | **Credit** | **Amount (€)** | **Factor** | **Product (€)** | **Product (€)** |
| 25321 | Bestrahlung mit einem Linearbeschleuniger bei bösartigen Erkrankungen oder bei raumfordernden Prozessen des zentralen Nervensystems, für das **erste** Zielvolumen, je Bestrahlungssitzung | 960 | 118.98 € | 20 | 2,379.6 | 960 | 118.98 | 28 | 3,331.44 | 594.9 |
| **Boost** | |  |  |  |  |  |  |  |  | |
| 25342 | Rechnerunterstützte Bestrahlungsplanung für die perkutane Bestrahlung mit individueller Dosisplanung für irreguläre Felder mit individuellen Blöcken, Viellamellenkollimator, nicht koplanaren Feldern und/oder 3-D-Planung | 4744 | 587.94 € | 0 | 0 | 4744 | 587.94 | 1 | 587.94 | 0 |
| 25343 | Zuschlag zur Gebührenordnungsposition 25342 für die rechnerunterstützte Hochpräzisionsbestrahlungsplanung (IMRT und/oder fraktionierte Stereotaxie), je Besrahlungsserie | 1245 | 154.30 € | 0 | 0 | 1245 | 154.30 | 1 | 154.30 | 0 |
| 25321 | Bestrahlung mit einem Linearbeschleuniger bei bösartigen Erkrankungen oder bei raumfordernden Prozessen des zentralen Nervensystems, für das erste Zielvolumen, je Bestrahlungssitzung | 960 | 118.98 € | 0 | 0 | 960 | 118.98 | 11 | 1,308.78 | 0 |
| **Other** | | | | | | | | | | |
| 25211 | Konsiliarpauschale bei bösartiger Erkrankung (Abschlussgespräch) | 1041 | 129.02 € | 1 | 129.02 | 1041 | 129.02 | 1 | 129.02 | 0 |
|  | | **Moderate HF: 20 x 3 Gy** | | | | **NF: 39 x 2 Gy (56 Gy main plan + 22 Gy boost)** | | | | **UHF:**  **5 x 5 Gy** |
| **EBM-**  **number** | **Description** | **Credits** | **Amount (€)** | **Factor** | **Product (€)** | **Credits** | **Amount (€)** | **Factor** | **Product (€)** | **Product (€)** |
| 40110 | Kostenpauschale für die Versendung bzw. den Transport eines Briefes und/oder von schriftlichen Unterlagen |  | 0.96 | 2 | 1.92 |  | 0.96 | 2 | 1.92 | 1.92 |
| 40111 | Kostenpauschale für die Übermittlung eines Telefaxes |  | 0.05 | 1 | 0.05 |  | 0.05 | 1 | 0.05 | 0.05 |
| 01601 | Individueller Arztbrief | 108 | 13.38 | 1 | 13.38 | 108 | 13.38 | 1 | 13.38 | 13.38 |
|  |  |  |  | **Sum** | **4,628.90** |  |  | **Sum** | **6,441.96** | **1,822.83** |

Table 8 – Revenues for hypofractionated (20 fractions and 5 fractions, respectively) and normofractionated (39 fractions) regimen for prostate cancer. Since ultra-hypofractionation is not a standard concept at most centers, the corresponding column on the right is shown in gray font.

EBM = uniform evaluation scale (Einheitlicher Bewertungsmaßstab); SIB = simultaneous integrated boost; IMRT = intensity-modulated radiotherapy; CT = computed tomography; HF = Hypofractionation; NF = Normofractionation; UHF = Ultrahypofractionation
